# MR-CISD investigation (including extensivity corrections) of Rydberg n-3 s and n-3p states of the smallest ionized HCl-water cluster

**DOI:** 10.1007/s00894-026-06912-3

**Published:** 2026-09-02

**Authors:** Mariana G. Bezerra, Elizete Ventura, Silmar A. do Monte

**Affiliations:** Departamento de Química, CCEN, Universidade Federal da Paraíba, João Pessoa, 58059-900 Brazil

**Keywords:** ionized HCl, MR-CISD calculations, Rydberg states

## Abstract

**Context:**

The [H_3_O(H_2_O)_3_]^+^Cl^−^ ion-pair is the smallest cluster of ionized HCl in water. It has been demonstrated experimentally that it can be formed in conditions compatible with the interstellar medium (ISM). It is also a precursor of another ion-pair, H_3_O^+^(MetOH)_3_Cl^−^, suggested as an important prebiotic species in the ISM. Aiming to help the detection of the former ion-pair several properties of its n-3 s and n-3p singlet and triplet Rydberg states have been studied through a highly correlated *ab-initio* method. The vertical excitation energies (ΔE_v_), oscillator strengths (*f*), dipole moments (μ), diradical character, participation ratio of natural transition orbitals (PR_NTO_), single-excitation character (Ω), charge-transfer numbers and the singlet–triplet gaps (ΔE_ST_) have been studied at the ground state (gs) geometry. The energies of the two lowest-lying dissociation channels have also been computed. Our highest-level results for ΔE_v_ of the n-3 s singlet states are between 6.63 and 6.65 eV, while for n-3p one has 7.40 to 7.74 eV. Previous TD-DFT results are ~ 0.84 to 0.95 eV lower, but our results nicely agree with available CASPT2 results for the n-3 s states. The brighter state is the 2nd n-3 s state, with *f* = 0.172 at the highest-level. While the gs is essentially a closed-shell state, consistent with the [H_3_O(H_2_O)_3_]^+^Cl^−^ ion pair, all n-3 s and n-3p singlet states have high diradical characters, consistent with their small ΔE_ST_ values and with their nature as charge-recombination (CR) states. For most of the cases the CR characters are consistent with a decrease of μ. A possible deactivation pathway for the n-3 s states, involving their optimized geometries and a four-states conical intersection, is discussed. Our highest-level results yield the ionic channel 0.46 eV lower than the neutral channel. Ω indicates dominant single-excitation character for all Rydberg states, while PR_NTO_ indicates multi-configurational character only for few n-3p states.

**Methods:**

The multi-reference configuration interaction with singles and doubles (MR–CISD) calculations (including size-extensivity corrections) have been employed. Size-extensivity corrections have only been applied for ΔE_v_ and for the energies of the dissociation channels. *f*, μ and ΔE_ST_ have been computed from the MR-CISD wavefunctions. PR_NTO_, Ω and the charge-transfer numbers (from which the nature of the excited states in terms of local, charge-transfer or CR states are obtained) have been computed from the one-electron transition density matrix. Three basis sets have been employed. The smallest one is the Pople’s 6–31+ +G**, while the second one is a mixed basis set based on the aug-cc-pVDZ and the dʹ-aug-cc-pVDZ (a reduced d-aug-cc-pVDZ basis set). The third one is also a mixed basis set, consisting of the aug-cc-pVDZ and the universal Rydberg basis set of Kaufman et al. The possible deactivation pathway for the n-3 s states has only been studied at the MR-CISD level with the smallest basis set.

**Supplementary Information:**

The online version contains supplementary material available at https://doi.org/10.1007/s00894-026-06912-3.

## Introduction

The dissociation (ionization) of strong acids and bases in aqueous solution is one of the most important processes in Chemistry. To understand this process at the molecular level for HCl, for instance, researchers have tried to figure out how many water molecules are required to ionize this acid. Experiments performed in He nanodroplets indicate that in ultracold temperatures (~ 0.4 K) four is the smallest number of water molecules to yield the dissociated acid, characterized by the solvent-shared ion pair H_3_O^+^(H_2_O)_3_Cl^−^ [1, 2]. The authors concluded that formation of this species is due to a sequential pickup of water molecules after the pickup of HCl [1], and explained the dissociation by a mechanism named aggregation-induced dissociation [2–4]. Several theoretical studies confirmed the number of four water molecules [4–16]. Tachikawa showed, by using direct ab initio molecular dynamics, the importance of the collision site between the fourth water molecular and a previously formed cyclic HCl(H_2_O)_3_ cluster for formation of a solvent-separated or contact ion-pair [12]. Lin and Paesani showed, using semi-empirical Born–Oppenheimer replica exchange molecular dynamics simulations of HCl(H_2_O)_n_ (with n = 4–10) clusters, that HCl exists in the dissociated form in all low-energy isomers [13]. These authors also showed, by studying the infrared spectra of HCl(H_2_O)_n_ (with n = 4–10 and 21) at 50 K from semi-empirical Born–Oppenheimer molecular dynamics simulations, the presence of vibrational modes associated with the excess proton [14]. Tudela and Marx confirmed, through ab initio path integral simulations, that four water molecules are required to dissociate HCl at low temperatures [15]. In larger clusters molecular dynamics studies using a semiempirical Hamiltonian show the prevailing presence of a water mediated interaction between the ions [17].

MP2, CCSD and DFT (BLYP) results indicate the solvent-shared ion pair H_3_O^+^(H_2_O)_3_Cl^−^ structure (of C_3_ symmetry) as the lowest energy isomer of the HCl(H_2_O)_4_ cluster [1, 18]. Vargas-Caamal et al*.* concluded that entropic effects at room temperature can change the mentioned smallest number of water molecules to five [19].

Refs. [1, 4], along with detection of water [20] and HCl [21, 22] in the molecular cloud Sagittarius B2 (Sgr B2) suggest a possible formation of the H_3_O^+^(H_2_O)_3_Cl^−^ ion pair in Sgr B2. Recently our group suggested a mechanism for formation of a methanol enriched ion pair, H_3_O^+^(MetOH)_3_Cl^−^, from the H_3_O^+^(H_2_O)_3_Cl^−^ ion pair in regions of Sgr B2 rich in methanol [18]. H_3_O^+^(MetOH)_3_Cl^−^ is suggested as an important prebiotic species in the interstellar medium [18], which increases the importance of the H_3_O^+^(H_2_O)_3_Cl^−^ species. Besides, the H_3_O^+^(H_2_O)_3_Cl^−^ cluster is the smallest neutral cluster for the study of the photochemistry of the chloride anion in an aqueous environment. In the atmosphere the HCl molecule is a very important Cl reservoir species, though essentially nonreactive with respect to gas-phase chemistry [23]. However, through heterogeneous reactions taking place in aerosol ice particles it can generate Cl_2_, which is then photolyzed to yield Cl radicals, one of the main ozone depleting species [23, 24].

The n-3 s and n-3p Rydberg states of the H_3_O^+^(H_2_O)_3_Cl^−^ cluster have been studied at the TD-DFT level (with the B3LYP functional) by Sobolewski and Domcke using the 6–31 + G** basis set extended through inclusion of additional diffuse s and p Gaussian functions on the oxygen atoms [25]. The authors also compared these states to those of the biradical and covalent forms. Later, the first two n-3 s singlet states have been studied at the CASSCF and CASPT2 levels using the 6–31 + + G** basis set [26].

In this manuscript the n-3 s and n-3p singlet and triplet Rydberg states of the H_3_O^+^(H_2_O)_3_Cl^−^ cluster have been studied at the multi-reference configuration interaction with singles and doubles (MR–CISD) level [27] (including size-extensivity corrections). Several properties of the mentioned excited states have been studied, as their vertical excitation energies, dipole moments, oscillator strengths, charge-transfer (CT) numbers and diradical character.

## Computational details

**Fig. 1:**
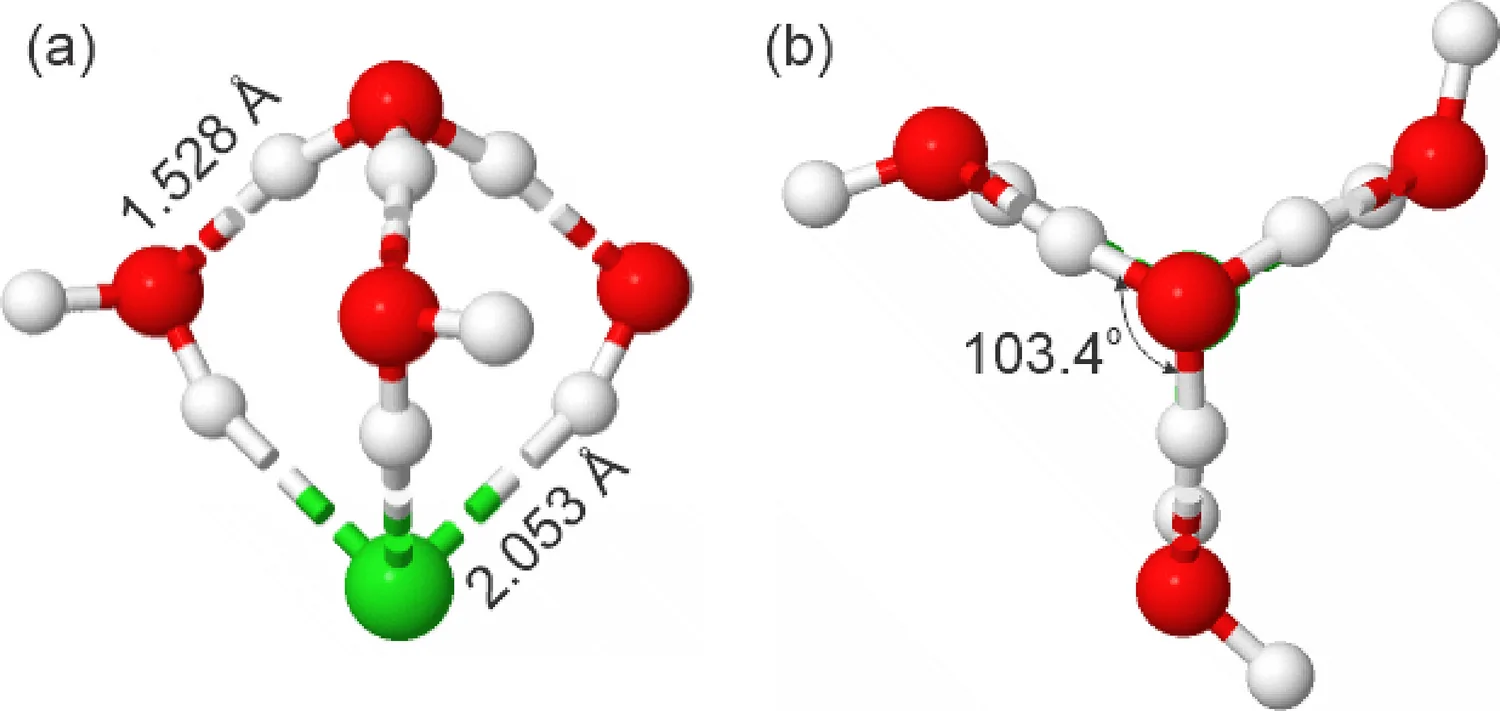
The structure of the H_3_O^+^(H_2_O)_3_Cl.^−^ ion pair studied in this work: (**a**) side-view; (**b**) top view. The structure shown has been optimized at the MP2/aug-cc-pVTZ level [18]

The geometry initially used for the excited states’ calculations is that from ref. [18], optimized at the MP2 level with the aug-cc-pVTZ basis set (see Fig. 1). As mentioned, the molecule has C_3_ symmetry. At the MCSCF level the excited configuration state functions (CSFs) obtained have been generated from single excitations from the 3 s and 3p orbitals of Cl to the 3 s and 3p Rydberg orbitals of the H_3_O^+^(H_2_O)_3_ moiety. These CSFs along with the ground state (closed shell) configuration yield 17 CSFs. For the calculation of the singlet states thirteen states, that is, the ground and the twelve Rydberg (n-3 s and n-3p) states have been included in the state-average MCSCF calculations, while in the case of the triplet states only the twelve triplet Rydberg states have been included. Thus, one has different sets of MCSCF orbitals for the singlet and triplet states. All calculations have been performed without any symmetry constraint. For both singlet and triplet states the same weight for the state-average MCSCF calculations is given to all states considered. Although the CSFs containing excitations from the 3 s(Cl) orbital do not have significant weights in the studied states, this orbital has been included in the restricted active space to improve the description of the 3 s Rydberg orbital.

The orbitals optimized at the MCSCF level have then been used for the subsequent MR-CISD and multi-reference averaged quadratic coupled cluster (MR–AQCC [28, 29]) calculations. At these levels the reference CSFs were generated as in the MCSCF calculations, that is, from single (3 s + 3p)(Cl) → 3 s + 3p (Rydberg) excitations along with the closed shell CSF. The final expansions for the MR–CISD and MR–AQCC wavefunctions include the reference CSFs along with those generated by applying single and double excitations from the internal (in this case restricted active plus doubly occupied) orbitals of the reference CSFs into all virtual orbitals. The K + L shells orbitals of Cl atom along with the K shell orbital of the O atom were kept frozen in all CI calculations. The interacting space restriction was applied for all MR-CISD calculations [30]. Size extensivity corrections have been employed using the a priori and a posteriori MR-AQCC [28, 29] and Pople [31] corrections (MR-CISD + P), respectively.

Three basis sets have been employed for the MCSCF and the MR-CISD calculations. The first one (named B1) is the 6–31 + + G** basis set [32–38], the same used in ref. [26]. The second one (named B2) is a mixed basis set consisting of the aug-cc-pVDZ basis set for H and Cl [39–41] and dʹ-aug-cc-pVDZ for the O atoms. This latter basis set has been obtained from the d-aug-cc-pVDZ [39, 40, 42] by removing the d function of the d-set. The third one (named B3) is also a mixed basis set, but now consisting of the aug-cc-pVDZ for the H, Cl and O atoms [39–41] and the universal Rydberg basis set of Kaufman et al*.* [43] centered on a dummy atom situated halfway between the O atom of the H_3_O moiety and the Cl atom (see Fig. 1). These three basis sets consist of 148, 216 and 272 Gaussian primitives, respectively.

The following properties have been computed: vertical excitation energies (ΔE_v_), the expectation values of r^2^ (< r^2^ > = < x^2^ > + < y^2^ > + < z^2^ >), oscillator strengths (*f*, for the singlet states), dipole moments (μ), diradical character (*y*), participation ratio of natural transition orbitals (PR_NTO_), single-excitation character (Ω), CT numbers and the singlet–triplet gaps (ΔE_ST_). ΔE_v_, < r^2^ >, μ and *y* have been computed at both MR-CISD and MR-AQCC levels. Only ΔE_v_ has also been computed at the MR-CISD + P level. The remaining properties (*f,* PR_NTO_*,* Ω, the CT numbers and ΔE_ST_) have only been computed at the MR-CISD level. *y* is computed from the overall number of unpaired electrons which, in its turn, is computed from the occupation numbers of the natural orbitals [44, 45]. The MR-AQCC calculations have only been performed for the first four singlet states with the B2 basis set. The MR–AQCC calculations of S_1_ to S_3_ showed intruder-states problems, that is, additional CSFs (not included in the reference space) that obtained an unreasonably large weight. To solve this problem these individual CSFs (up to sixteen) were included in the reference space as well.

All MR-CISD and MR-AQCC calculations have been carried out with the COLUMBUS program system [46–49], with integrals calculated by the Dalton program [50]. All geometry optimizations and linear interpolations performed with COLUMBUS use the natural internal coordinates (also named natural valence coordinates) defined by Fogarasi *et. al*.[51]. The one-electron transition density matrix (1TDM) between the ground and the excited singlet states, taken from COLUMBUS, have been used to compute PR_NTO_ [52–54], Ω [52, 55, 56] and the CT numbers [53–55, 57–62] (see also eqs.(1) and (2) from ref. [57], eqs.(5), (7) and (15) from ref. [59] and eqs.(1), (2) and (3) from ref. [62]). The PR_NTO_ values indicate how many pairs of NTOs are participating and, thus, how many configurations are necessary to describe each excited state [52, 57, 59]. The CT numbers are used to assess the nature of the excited states in terms of the following contributions concerning the Cl and [(H_2_O)_3_H_3_O] fragments: (i) local excitations (LE), (ii) charge-recombination (CR) excitations (Cl^−^ → [(H_2_O)_3_H_3_O]^+^, (iii) charge-transfer (CT) excitations ([(H_2_O)_3_H_3_O]^+^ → Cl^−^) [57, 62]. The wavefunction descriptors PR_NTO_, Ω and the CT numbers were computed using the TheoDORE 3.1.1 program package [57]. All MP2 calculations have been performed with the g09 software [63].

The energies of the two lowest lying singlet dissociation channels associated with release of Cl have also been computed at the MR-CISD/MR-CISD + P levels with the three basis sets B1, B2 and B3. For these calculations the excited CSFs at the MCSCF level have been generated from single excitations from the 3p orbitals of Cl to the 3 s Rydberg orbitals of the [(H_2_O)_3_H_3_O] moiety. These CSFs along with the closed shell configuration yield 4 CSFs. The first four singlet states have been included in the state-average MCSCF calculations. As previously, the same weight is given to all (four) states considered. Again, at the MR-CISD/MR-CISD + P level the reference CSFs were generated as in the MCSCF calculations, and the same approach as before has been used to generate the final MR-CISD expansion. The supermolecule approach has been used for the calculations involving the dissociation channels. In the case of the neutral and ionic channels the geometries of the [H_3_O(H_2_O)_3_]• and [H_3_O(H_2_O)_3_]^+^ species have been optimized at the UMP2 and MP2 levels, respectively, using the aug-cc-pVTZ basis set. Then the Cl atom was placed at 50 Å from the O atom of the H_3_O moiety. For the calculation of the energy of the ionic channel the residual Coulomb energy between the charged species (0.29 eV at 50 Å) was further discounted from the potential energy of the supermolecule. In the case of supermolecule calculation with the B3 basis set the dummy atom is situated at the same point as in the calculation using the optimized ground state geometry, but only with respect to the H_3_O(H_2_O)_3_ moiety. The same MR-CISD expansion used for the calculations of the dissociation channels has been used for full geometry optimization attempts of S_1_ to S_3_ with B1 and B2, to check whether at least one of the n-3 s states is bonded. This same MR-CISD expansion has also been used to optimize the ground state geometry (with B1 and B2), for a more consistent comparison between this latter geometry and that of the n-3 s state. The starting geometry for the optimization of the n-3 s state has been obtained from the optimized ground state geometry simply moving the fragments to 20 Å from each other. This came from several attempts to generate a good starting geometry. This latter MR-CISD expansion has also been used for a potential energy curve calculation for S_0_ to S_3_ with B1 along linearly interpolated geometries between the optimized ground state and the S_1_/S_2_/S_3_ geometries.

The sizes of the MR-CISD expansions vary from ~ 11 to ~ 197 millions of CSFs, for the calculations involving the dissociation channels with the B1 basis set and for the vertical calculations with the B3 basis set, respectively.

## Results and discussion

Due to the near degeneracy of the 3p(Cl) lone pairs and the 3p(Rydberg) orbitals which are almost perpendicular to the C_3_ axis (see Fig. 1) several pairs of singlet and triplet states have been considered as single states and have been renamed as S_i_’ and T_i_’ (respectively), with i = 0 to 7, as explained in Table 1 (for B3). The corresponding pairs for B1 and B2 are shown in Table S1. These degeneracies have also been mentioned in ref. [25]. For the sake of consistency, the remaining states have also been renamed according to the same nomenclature. The mentioned degeneracies affect the way the studied properties are computed. ΔE_v_, < r^2^ >, μ, *y*, PR_NTO_*,* and Ω are computed as the averages between the corresponding pairs of states, when it applies (see Table 1). On the other hand, each *f* is computed as the sum of the values of the corresponding pairs of states, when it is the case. Finally, the singlet–triplet gaps (ΔE_ST_) are computed between the S_i_’ and T_i_’ states with the most similar configurations, also given in Table 1.

**Table 1:** Pairs of singlet and triplet states taken as single states (for B3), due to the degeneracies mentioned in the text. The pairs of singlet and triplet states used for the calculation of ΔE_ST_ are also given

singlet states	combined states		final state
	S_0_	–	S_0_ʹ
	S_1_	–	S_1_ʹ
	S_2_	S_3_	S_2_ʹ
	S_4_	S_5_	S_3_ʹ
	S_6_	S_7_	S_4_ʹ
	S_8_	S_9_	S_5_ʹ
	S_10_	–	S_6_ʹ
	S_11_	S_12_	S_7_ʹ
triplet states	combined states		final state
	T_1_	–	T_1_ʹ
	T_2_	T_3_	T_2_ʹ
	T_5_	T_6_	T_3_ʹ
	T_4_	T_9_	T_4_ʹ
	T_7_	T_8_	T_5_ʹ
	T_10_	–	T_6_ʹ
	T_11_	T_12_	T_7_ʹ
pairs of states used for the calculation of ΔE_ST_	S_1_ʹ/T_1_ʹ		
	S_2_ʹ/T_2_ʹ		
	S_3_ʹ/T_3_ʹ		
	S_4_ʹ/T_4_ʹ		
	S_5_ʹ/T_5_ʹ		
	S_6_ʹ/T_6_ʹ		
	S_7_ʹ/T_7_ʹ		

In Table 2 the computed properties ΔE_v_, < r^2^ >, μ, *y*, *f,* PR_NTO_*,* Ω, and ΔE_ST_ are given at the MR-CISD level for the first six excited singlet states. < r^2^ > has also been computed for the ground and triplet states for comparison, while μ and *y* of the ground state are also computed for comparison. The main weights of the dominating configurations are also given. As mentioned previously only ΔE_v_ has also been computed at the MR-CISD + P level. In Table 2 only the results obtained with B3 are shown. The corresponding results for B1 and B2, along with their analysis, are given in Table S2 and in Section 4.1 of SI.

**Table 2:** ΔE_v_, < r^2^ >, μ, *y*, *f,* PR_NTO_*,* Ω, and ΔE_ST_ computed at the MR-CISD level for the first six excited singlet states, computed with B3. < r^2^ > values for the ground and triplet states are also given, while μ and *y* of the ground state are also given

state	ΔE_v_^a^	main configurations^b^	< r^2^ > ^c^	μ^d^	*y*	*f* (× 10^2^)	PR_NTO_	Ω	ΔE_ST_^a^
S_0_ʹ	0.00	0.81cs^d^	84.68	4.71	0.034	–-	–-	–-	–-
S_1_ʹ(1)^f^	6.01 (6.63)^g^	0.83n_z_3s	133.77	1.20	2.033	5.33	1.001	0.951	0.09
S_2_ʹ(2)^f^	6.02 (6.65)^g^	0.72n3s	137.18	1.11	2.033	17.21	1.004	0.953	0.09
S_3_ʹ(3)^f^	6.69 (7.40)^g^	0.66n_z_3p	169.55	3.73	2.078	1.42	1.229	0.954	0.02
S_4_ʹ(4)^f^	6.72 (7.44)^g^	0.58n3p + 0.16n_z_3p	169.83	0.78	2.294	0.90	1.928	0.955	0.00
S_5_ʹ(5)^f^	6.79 (7.49)^g^	0.77n3p	170.89	0.30	2.176	0.90	2.202	0.952	0.04
S_6_ʹ(6)^f^	7.00 (7.71)^g^	0.65n_z_3p_z_	191.06	0.91	1.981	0.07	1.213	0.947	0.03
S_7_ʹ(7)^f^	7.00 (7.74)^g^	0.72n3p_z_	194.71	1.27	2.034	0.66	1.008	0.955	0.03
T_1_ʹ(1)^f^	5.92 (6.55)^g^	0.82n_z_3s	133.40	–-	–-	–-	–-	–-	–-
T_2_ʹ(2)^f^	5.93 (6.55)^g^	0.83n3s	133.84	–-	–-	–-	–-	–-	–-
T_3_ʹ(3)^f^	6.67 (7.37)^g^	0.57n_z_3p + 0.26n3p	165.75	–-	–-	–-	–-	–-	–-
T_4_ʹ(4)^f^	6.72 (7.41)^g^	0.53n3p + 0.19n_z_3p	165.92	–-	–-	–-	–-	–-	–-
T_5_ʹ(5)^f^	6.75 (7.39)^g^	0.82n3p	166.16	–-	–-	–-	–-	–-	–-
T_6_ʹ(6)^f^	6.97 (7.64)^g^	0.81n_z_3p_z_	188.42	–-	–-	–-	–-	–-	–-
T_7_ʹ(7)^f^	6.97 (7.71)^g^	0.82n3p_z_	189.35	–-	–-	–-	–-	–-	–-

^a^values in eV; ^b^n and 3p stand for the (n_x_,n_y_) and (3p_x_,3p_y_) pairs, respectively; ^c^values in au (a_0_^2^); ^d^values in Debye; ^e^cs = closed shell; ^f^The number in parenthesis identify the S/T pair with the most similar configurations, used to computed ΔE_ST_; ^g^values at the MR-CISD + P level; weights smaller than 0.1 have not been included

The < r^2^ > values of the excited singlet states are significantly larger than that of the ground state, for the three basis sets, which is consistent with the Rydberg nature of the excited states (see Tables 2 and S2). As shown in Table 2 for the ground state it is 84.68a_0_^2^, while for the excited states they vary from 133.77 to 194.71a_0_^2^. The < r^2^ > values of the triplet states vary in similar ranges (see Tables 2 and S2).

The excitation energies obtained at the MR-CISD/B3 level for the singlet states are in the range from 6.01 to 7.00 eV (see Table 2). The size-extensivity correction (MR-CISD + P results in Table 2) increases the ΔE_v_ values considerably, in a range from 0.62 to 0.74 eV. As in the case of the TD-DFT/B3LYP results in ref. [25] the n-3 s and n-3p states are well separated, at both MR-CISD/B3 and MR-CISD + P/B3 levels. While at the TD-DFT level the separation is 0.60 eV, at the MR-CISD/B3 level it is slightly larger, 0.67 eV (see Table 2). At the MR-CISD + P/B3 level it increases to 0.75 eV.

Concerning the ΔE_v_ values of the n-3 s singlet and triplet states the three basis sets studied in this work yield similar results, at both MR-CISD and MR-CISD + P levels (compare Tables 2 and S2). At the MR-CISD level the n-3p singlet states are in a range from 6.69 to 7.00 eV. At the MR-CISD + P level these values increase 0.41 to 0.74 eV (see Table 2). The range obtained for the n-3p triplet states at the MR-CISD level is 6.67 to 6.97 eV. At the MR-CISD + P level these values increase 0.62 to 0.74 eV (see Table 2).

Our highest-level results for ΔE_v_ of the n-3 s singlet states (S_1_’ and S_2_’), obtained at the MR-CISD + P level with B3, are 6.63 and 6.65 eV. The basis set effect is small for the n-3 s states (see Tables 2 and S2). As shown in Table S2 the MR-AQCC calculations have only been performed for S_1_’ and S_2_’ with B2. Assuming the same effect for B2 and B3, as compared to the results at the MR-CISD + P level, our highest-level results for S_1_’ and S_2_’ would change to 6.76 and 6.78 eV. In the case of T_1_’ and T_2_’ (the n-3 s triplet states) our most accurate results are 6.55 eV for both states (see Table 2).

In the case of the n-3p singlet states (S_3_’ to S_7_’) our highest-level results (again obtained at the MR-CISD + P level with the B3 basis set) are between 7.40 and 7.74 eV. For the n-3p triplet states (T_3_’ to T_7_’) our highest-level results are between 7.37 and 7.71 eV, a range very close to that obtained for S_3_’ to S_7_’.

The basis set effect is also important for the main configurations of both singlet and triplet states studied in this work, as discussed in Section 4.1 of SI (see also Tables 2 and S2). For B3 one has n_z_ 3 s and n3s as the sole main configurations in S_1_’ and S_2_’, respectively. In the case of T_1_’ and T_2_’ the main configurations are n_z_ 3 s and n3s, respectively (see Table 2).

S_3_’ and S_5_’ are characterized by only one main configuration (n_z_3p and n3p, respectively), for the three basis sets (see Tables 2 and S2). S_6_’ and S_7_’ are the n-3p singlet states whose main configurations change more with the basis set, as discussed in Section 4.1 of SI (see also Tables 2 and S2). For B3 their main configurations are n_z_3p_z_ and n3p_z_, respectively (see Table 2). The main configurations of S_4_’ (n3p and n_z_3p) do not change with the basis set (see Tables 2 and S2).

In the case of the n-3p triplet states the main configurations of T_3_’ to T_5_’ change with the basis set, as discussed in Section 4.1 of SI (see also Tables 2 and S2). For the n3p configuration is the dominant one for T_4_’ and T_5_’, but for the latter state it becomes much more important. For T_3_’ and T_4_’ the main configurations n_z_3p and n3p are swapped (see Table 2).

It is important to mention that some pairs of singlet states are almost degenerate but differ mainly in the values of *f*. For instance, in the case of S_1_′ and S_2_′ states with B1 one has similar values of the dipole moments (0.41 and 0.77 D, see Table S2) but significantly different values of *f* (0.0611 and 0.1988, see Table S2). The same holds for the S6′ and S7′ states with B1 (see Table S2). For B3 one has the same behavior for both pairs of states (see Table 2). On the other hand, for B2 one has the same behavior for the S_1_′ and S_2_′ states but not for the S_6_′ and S_7_′ states (see Table S2). For both pairs of quasi-degenerate states computed with the three basis sets one has different numbers of combined states (see Tables 1 and S1), which can explain the mentioned behavior.

S_2_’ is the brightest state, followed by S_1_’, for the three basis sets, in agreement with the TD-DFT/B3LYP results from ref. [25] (see Tables 2 and S2). As discussed before, both are n-3 s states, also in agreement with ref. [25]. Our highest-level *f* values for these states are *f* = 0.1721 and 0.0533, respectively, while for the n-3p states the *f* values are between 0.07 and 0.0142 (see Table 2). Higher *f* values for n-3 s (or n-4 s), as compared to the values for n-3p (or n-4p) states have also been observed for MR-CISD calculations of several other chlorinated systems [64–73].

**Fig. 2:**
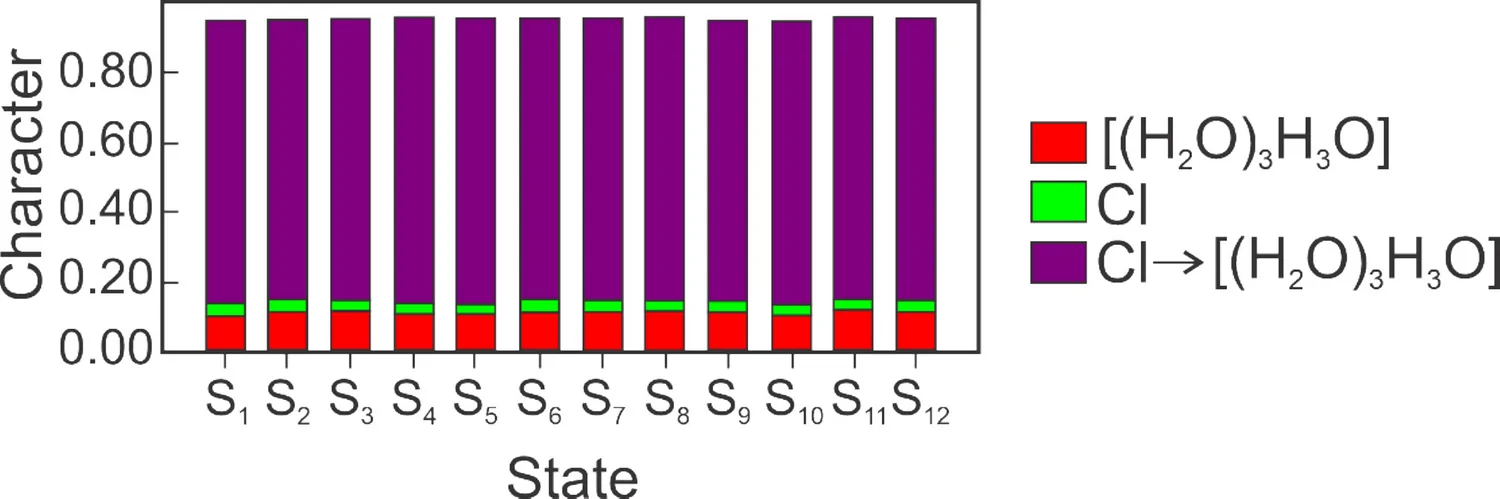
Fragment-based analysis of the characters of the excited singlet states of the [H_3_O(H_2_O)_3_]^+^ Cl^−^ ion-pair, computed at the MR-CISD level with the aug-cc-pVDZ(H, Cl,O)/Universal Rydberg(dummy) basis set

The diradical character is quantified by the *y* parameter (see Eq. (14) from ref. [44]). It is zero for an idealized closed-shell molecule and two for a pure diradical or zwitterionic state, while intermediate values characterize diradicaloid systems [44]. It is clear from Tables 2 and S2 that the ground state corresponds to an essentially closed-shell system, while all excited states are approximately diradical or zwitterionic states (for all three basis sets). In the closed-shell electronic configuration the three lone pairs of the Cl atom (that is, the HOMO-2, HOMO-1 and the HOMO orbitals) are doubly occupied, characterizing the presence of the chloride anion and thus leading to an ion-pair. Although the ion pair studied in this work is normally represented as H_3_O^+^(H_2_O)_3_Cl^−^, a significant positive charge transfer within the [H_3_O(H_2_O)_3_]^+^ moiety is expected, as the LUMO orbital of the ion pair has a relatively large contribution of a diffuse 3 s Rydberg orbital mainly localized on the (H_2_O)_3_ fragment, in line with the result from ref. [25]. This charge reorganization has already been identified in the [H_3_O(H_2_O)_3_]^+^ moiety [74]. Thus, [H_3_O(H_2_O)_3_]^+^Cl^−^ would be a better representation of the mentioned ion pair. However, as this analysis is beyond the scope of this work, it will not be further explored. The excited states are better described as approximate diradical states instead of zwitterionic states. Such description is consistent with: (i) the analysis of the CT numbers of the excited states; (ii) the smaller dipole moments of the excited states (as compared to that of the ground state), obtained with B3; (iii) the small values of ΔE_ST_, obtained at the MR-CISD level (for the sake of consistency with the *y* values, computed at the same level). Items (i) and (ii) will be discussed later (Figs. 2 and S1).

**Fig. 3:**
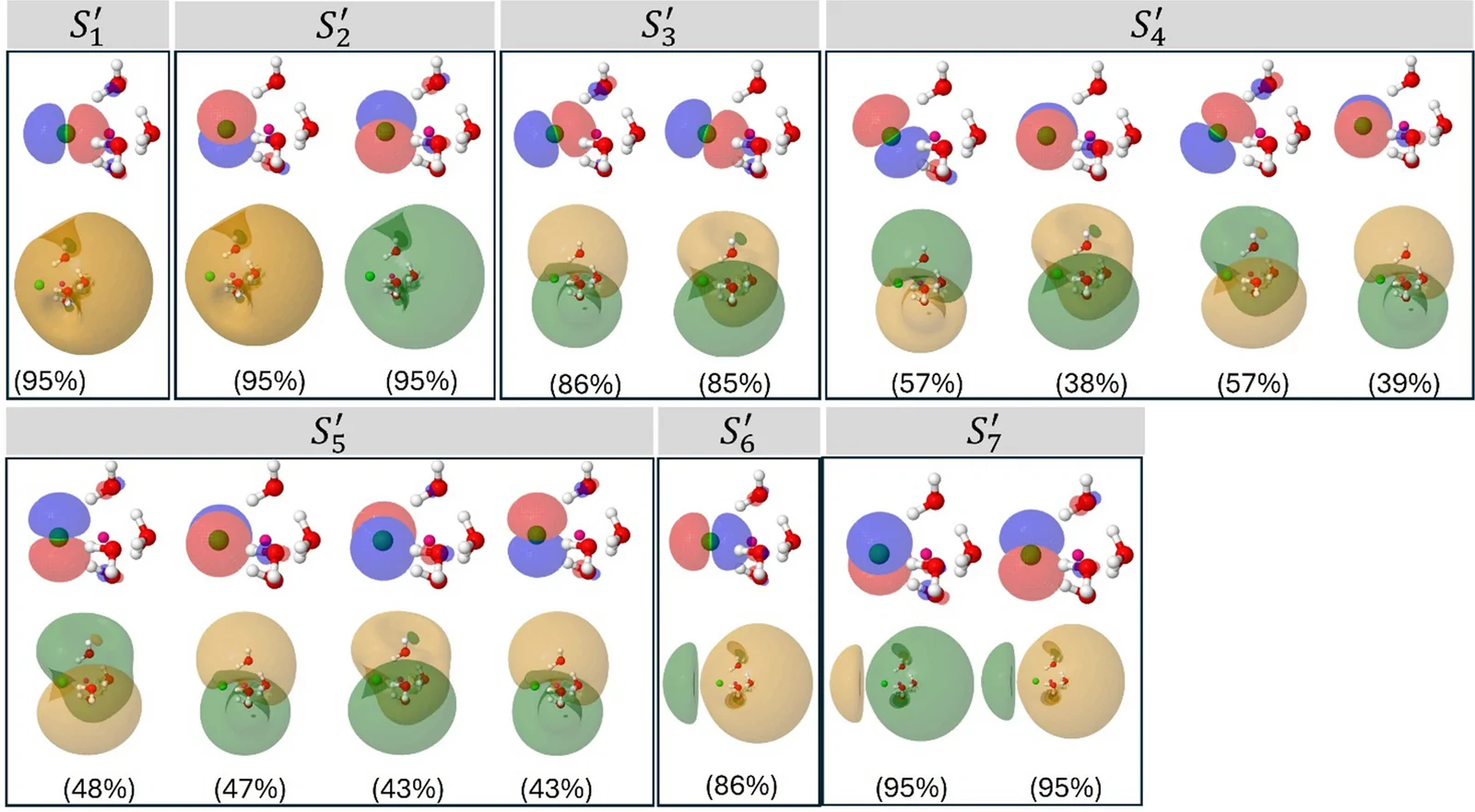
Dominant pairs of NTOs (isovalues: ± 0.04 and ± 0.012 a.u. for the hole and electron NTOs, respectively), along with their contributions, obtained at the MR-CISD level with B3 for the S_1_’ − S_7_’ states of the ion pair studied in this work. The hole is shown on the bottom and the electron at the top. Refer to Table 1 for the original singlet states contained in S_i_’(i = 1 to 7)

Ω, which measures whether an excited state can be described as a linear combination of single excitations or whether higher excitations are needed (see Eq. (7) and Fig. 4 of ref. [59]), is given in Tables 2 and S2 for all studied singlet states. It is clear from these tables that these states are essentially singly excited states (as Ω is close to 1), which is consistent with the nature of the Rydberg states. Figure 7 from ref. [75] suggests that Pople correction is very important to decrease the errors of ΔE_v_ for predominantly singly excited states (that is, those with Ω > 0.75 [75]). Figure 2 shows characters of the excited singlet states, obtained from the analysis of the CT numbers (see eqs.(2) and (15) from refs [57]. and [59], respectively) with respect to the Cl and the [(H_2_O)_3_H_3_O] fragments. In this plot only the results obtained with B3, at the MR-CISD level, are shown. The corresponding plot with B1 and B2 is shown in Figure S1. It is clear from Figs. 2 and S1 that one has a large CR of the type Cl^−^ → [H_3_O(H_2_O)_3_]^+^ for all studied excited singlet states. Only for B3 this CR is consistent with the decrease of the dipole moments of all excited singlet states, as compared to that of the ground state (see also Tables 2 and S2 and Section 4.1 of SI).

**Fig. 4:**
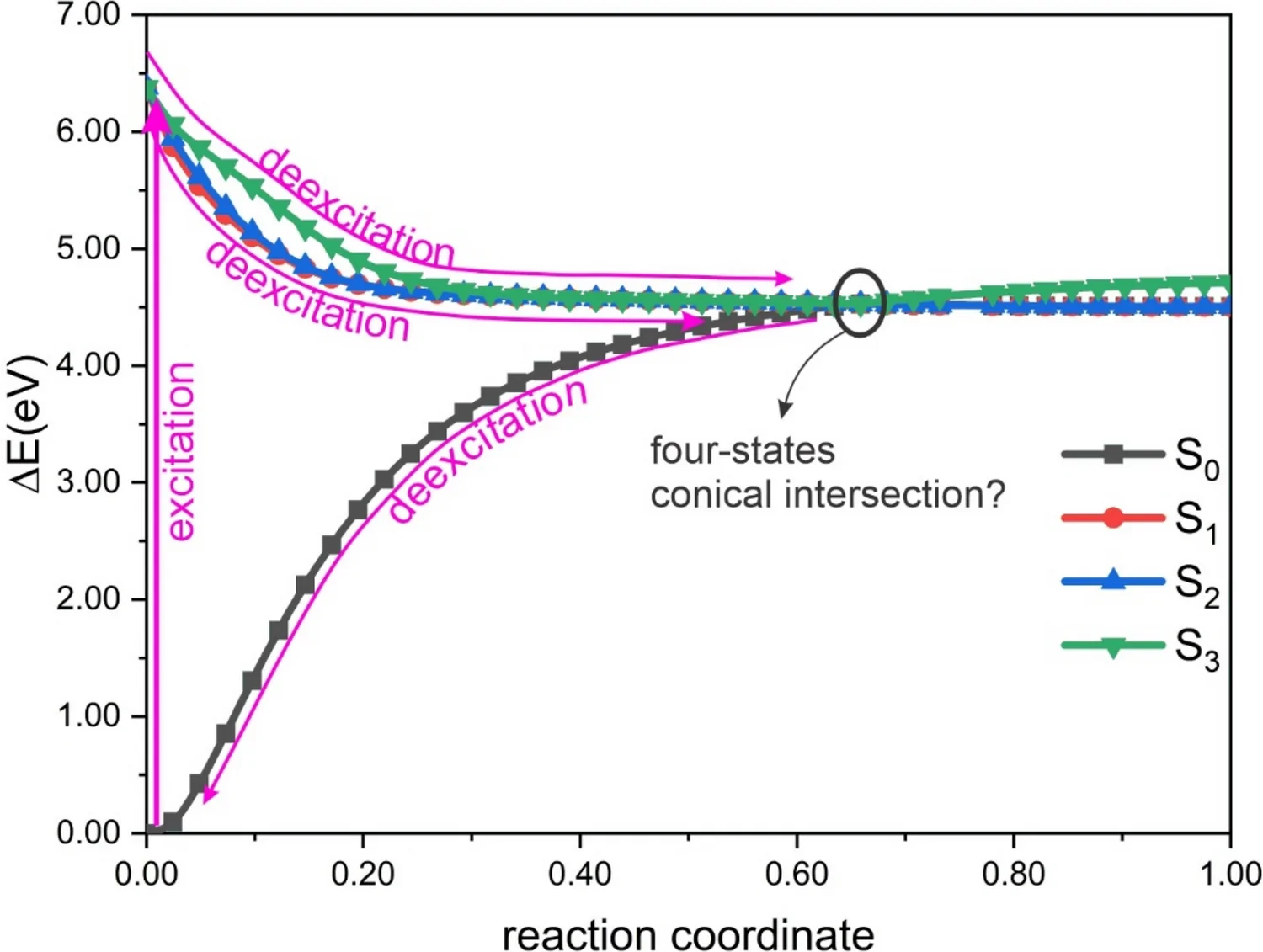
Potential energy curves at the MR-CISD/B1 level for S_0_ to S_3_ along the linear interpolated pathway from the S_0_ minimum to the stationary point for S_1_/S_2_/S_3_. The deactivation pathways discussed in the text are also shown

As aforementioned, the PR_NTO_ parameter measures the multiconfigurational character of a given state, which is equivalent to the number of pairs of NTOs participating in each excited state [52, 57, 59]. Most of the obtained values are relatively close to the integer values of 1 and 2 (see Table 2). According to the values of PR_NTO_ S_1_’, S_2_’, S_3_’, S_6_’ and S_7_’ are predominantly single configurational states, while S_4_’ and S_5_’ are multi-configurational (though singly excited) states (see Table 2). The hole and electron NTOs for each studied excited singlet state, computed at the MR-CISD level with B3, are given in Fig. 3. For the other basis sets the plots are very similar and thus are not shown. All pairs of NTOs support the previous assignment, that is, all studied excited singlet states are CR states (compare Figs. 2 and 3). Through comparison between the NTOs and the MOs it is clear that they are very similar, which indicates that the transitions are well described as simple MO transitions (see Fig. 4 from ref. [59]).

### Energetics of the two lowest lying dissociation channels

The two lowest lying dissociation channels of the [H_3_O(H_2_O)_3_]^+^Cl^−^ ion pair yield the isolated ions ([H_3_O(H_2_O)_3_]^+^ and Cl^−^) and the neutral fragments, [H_3_O(H_2_O)_3_] and Cl. In Table 3 the energies of these two channels are compared to that of the bonded ion pair, for B3. The results with B1 and B2 are given in Table S3 and discussed just after this table, in the SI. The studied ion pair is strongly bonded by 4.45 and 4.73 eV, at the MR-CISD and MR-CISD + P levels, respectively. However, it is important to point out that the inclusion of the so-called “tight”-d functions in the aug-cc-pVDZ basis set of molecules containing second row elements (forming the aug-cc-pV(D + d)Z basis set of Dunning *et. al*. [76]) can change the dissociation energy by up to 5 kcal/mol (~ 0.22 eV). Our highest-level result, 4.73 eV (see Table 3), is similar to the values of other ion pairs containing Cl^−^, that is, 4.65 (H_2_C^+^H•••Cl^−^ [68]) and 4.72 (HFC^+^H•••Cl^−^ [72]).

**Table 3:** Energies of the two dissociation channels (in eV) compared to that of the bonded ion pair, computed with B3. The zero-point energy (ZPE) corrections, computed at the MP2/aug-cc-pVTZ level, have been included

dissociation channel	MR-CISD	MR-CISD + P
[H_3_O(H_2_O)_3_]^+^ + Cl^−^	4.45	4.73
[H_3_O(H_2_O)_3_] + Cl	4.65	5.19

For all three basis sets and at the two computational levels shown in Tables 3 and S3 the ionic channel has a smaller energy than that of the neutral channel. At the MR-CISD/B3 level the difference is 0.20 eV. As can be seen from Tables 3 and S3 this difference is almost independent of the basis set at the MR-CISD level. At the MR-CISD + P level the differences are considerably larger, especially for B2 and B3 (see Tables 3 and S3). The corresponding difference at this level is 0.46 eV (see Table 3). Our highest-level result, 0.46 eV, is in very good agreement with the value of 0.44 eV derived from the very accurate value of 2.92 eV for the vertical electron attachment energy (VEAE) of the [H_3_O(H_2_O)_3_]^+^ cation, computed with the ab initio electron propagator D3, OVGF, P3 and P3 + methods with the d-aug-cc-pVTZ basis set [77]. The value of 0.44 eV has been obtained from the VEAE along with the relaxation energy of the [H_3_O(H_2_O)_3_] radical, the difference between the ZPEs of the cation and radical and the electron affinity of Cl, 3.63 eV. These three later values have been computed at the MP2/aug-cc-pVTZ level, and the later value is in excellent agreement with the experimental electron affinity of Cl, 3.62 eV [78]. The relaxation energy of the [H_3_O(H_2_O)_3_] radical is the difference between the energy of the radical at the geometry of the cation and at its own geometry.

Due to the highest *f* value for one of the n-3 s singlet states an important question arises, that is, whether they are bonded or not. To answer this question, we performed additional full geometry optimizations at the MR-CISD level (with B1 and B2) of the S_1_ to S_3_ states starting at a large distance (20 Å) between the fragments. The results are given in Figure S2 and discussed in Section 6 of SI. As explained before, the same CI space used to compute the energies of the dissociation channels have been used. Due to the quasi-degeneracy between the n-3 s states a relatively loose set of criteria for the parameters of the gradient program has been used, as explained in the SI. Besides, also due to the quasi-degeneracy mentioned a frequency calculation to check if the obtained structures are minima has not been performed.

### Possible deactivation pathways for the n-3 s states

The potential energy curves (at the MR-CISD level) for S_0_ to S_3_ along the linear interpolated pathway from the S_0_ minimum to the stationary point for S_1_/S_2_/S_3_ are given in Fig. 4. The results shown in this plot have been obtained with B1. Although we have also attempted to compute the mentioned curves with B2 we only succeeded for very few points, due to convergence problems at the MCSCF or MR-CISD level. As can be seen from Fig. 4 the n-3 s states (S_1_ to S_3_) can be deactivated during the Cl release, regenerating the ground state through a possible four-states conical intersection between S_0_ and the n-3 s states [79]. From Fig. 4 this intersection should take place at a value of ~ 0.658 for the reaction coordinate. This value corresponds to a distance of ~ 14.4 Å between the O atom of H_3_O and the Cl atom.

Although the plot in Fig. 4 shows potential deactivation pathways for the n-3 s states, they are not reported in the nonadiabatic dynamic calculations starting on S_1_ performed through the Full Multiple Spawning (FMS) method at the CASSCF/B1 level [26]. Instead, one has photochemical reaction channels involving dissociation of H_3_O, either direct (73%) or indirect, that is, after hydrogen transfer (27%) [26].

## Conclusions

Highly correlated MR-CISD calculations, including extensivity Pople’s corrections (MR-CISD + P), have been performed for the smaller cluster of ionized HCl, [H_3_O(H_2_O)_3_]^+^Cl^−^. Three basis sets (named B1, B2 and B3) have been used to study several properties of the n-3 s and n-3p singlet and triplet Rydberg states at the ground state geometry. B1 is simply the 6–31 + + G** basis set. B2 is a mixed basis set consisting of aug-cc-pVDZ for H and Cl and dʹ-aug-cc-pVDZ for the O atoms, where dʹ means removal of the d function of the d-set of the d-aug-cc-pVDZ basis set. B3 is also a mixed basis set, but now consisting of the aug-cc-pVDZ for the H, Cl and O atoms and the universal Rydberg basis set of Kaufman et al*.* [43] The following properties have been studied at the ground state geometry: vertical excitation energies (ΔE_v_), < r^2^ >, oscillator strengths (*f*, for the singlet states), dipole moments (μ), diradical character (*y*), participation ratio of natural transition orbitals (PR_NTO_), single-excitation character (Ω), charge-transfer (CT) numbers, the singlet–triplet gaps (ΔE_ST_) and the main configurations of the studied states. ΔE_v_, < r^2^ >, μ and *y* have been computed at both MR-CISD and MR-AQCC levels, but at this latter level only for the n-3 s singlet states with B2. Only ΔE_v_ has also been computed at the MR-CISD + P level. The remaining properties (*f,* PR_NTO_*,* Ω, the CT numbers and ΔE_ST_) have only been computed at the MR-CISD level.

The energetics of the two lowest lying dissociation channels have also been studied at the MR-CISD/MR-CISD + P levels with the three mentioned basis sets. These are the neutral ([H_3_O(H_2_O)_3_] + Cl) and the ionic ([H_3_O(H_2_O)_3_]^+^ + Cl^−^) channels. The n-3 s states have also been optimized with B1 and B2. Potential energy curves for the 3 s states (at the MR-CISD/B1 level), along linearly interpolated structures connecting the ground state and the 3 s optimized geometries, have also been computed.

The ΔE_v_ values of the n-3 s singlet and triplet states computed with the B1, B2 and B3 yield similar results, at both MR-CISD and MR-CISD + P levels. The largest differences are observed for the highest n-3p singlet and triplet states, but for B1 with respect to the other two basis sets. Our highest-level results for ΔE_v_ of the n-3 s singlet states, obtained at the MR-CISD + P/B3 level, are 6.63 and 6.65 eV, while at the TD-DFT/B3LYP level they are much lower, at ~ 5.70 eV [25]. The results at the CASPT2 level with B1 are 6.46 and 6.52 eV [26], in very good agreement with our MR-CISD + P/B1 result (6.57 eV). Including the more accurate extensivity corrections at the MR-AQCC/B2 level the ΔE_v_ values of the n-3 s states increase slightly (0.13 eV), as compared to the results at the MR-CISD + P/B2 level. In the case of the n-3 s triplet states our most accurate results are 6.55 eV.

Our highest-level results for ΔE_v_ of the n-3p singlet states are in a range from 7.40 to 7.74 eV. Small differences between the results obtained with B2 and B3 have been observed. At the TD-DFT/B3LYP level the corresponding range is from 6.30 to 6.90 eV [25], again much lower than our MR-CISD + P results. For the n-3p triplet states our highest-level results are in a range very close to that obtained for the singlet states. In general, the main configurations of the n-3 s states are less basis set dependent than those of the n-3p states.

All n-3 s and n-3p Rydberg singlet states have high diradical characters (*y*), consistent with their large charge-recombination (CR) characters (Cl^−^ → [H_3_O(H_2_O)_3_]^+^), as obtained from the CT numbers. On the other hand, the ground state structure is essentially a closed shell structure, consistent with the [H_3_O(H_2_O)_3_]^+^Cl^−^ representation, as expected. The diradical character of the excited states is consistent with small singlet–triplet gaps. For most of the cases an increase in the diradical character, from the ground to the excited singlet states, is accompanied by a decrease of μ. The Ω values of all studied excited singlet states are close to 1, which indicate essentially singly excited states, in line with the nature of Rydberg states. The values obtained for the PR_NTO_ parameter with B2 and B3 indicate that most of the studied Rydberg states are single configurational states, but two (n-3p) are multi-configurational (though singly excited) states.

In agreement with what has been observed for several Cl-containing molecules [64–73], comparing only n-3 s and n-3p singlet states, the state with the higher *f* value is in the former set. Therefore, it is important to study possible photochemical/photophysical pathways starting in the Franck–Condon region of the n-3 s states. In this context, potential energy curves for S_0_ to S_3_ (at the MR-CISD/B1 level) along linearly interpolated structures connecting the ground state and the n-3 s optimized geometries suggest a possible deactivating pathway of the n-3 s states to the ground state via a four-states conical intersection (see Fig. 4).

All our MR-CISD/MR-CISD + P results yield the ionic dissociation channel ([H_3_O(H_2_O)_3_]^+^ + Cl^−^) lower than the neutral ([H_3_O(H_2_O)_3_] + Cl) channel. Our highest-level result, 0.46 eV (see Table 3), is in very good agreement with the value of 0.44 eV derived from the vertical electron attachment energy (VEAE) of the [H_3_O(H_2_O)_3_]^+^ cation along with the relaxation energy of the [H_3_O(H_2_O)_3_] radical, the ZPEs of the cation and the radical and the electron affinity of Cl. VEAE has been computed from ab initio electron propagator methods with the d-aug-cc-pVTZ basis set [77], while the later three quantities have been computed at the MP2/aug-cc-pVTZ level.

## Supplementary Information

Below is the link to the electronic supplementary material.

Supplementary file1 (DOCX 327 KB)

## Data Availability

No datasets were generated or analysed during the current study.
