# Supplementary material for: MR-CISD investigation (including extensivity corrections) of Rydberg n-3 s and n-3p states of the smallest ionized HCl-water cluster
Source: J Mol Model. 2026 Sep 2;32(9):345. doi: 10.1007/s00894-026-06912-3 (PMC13538156; doi:10.1007/s00894-026-06912-3)
Supplement: Supplementary file 1 [file 894_2026_6912_MOESM1_ESM.docx]

**Supporting Information**

MR-CISD Investigation (including extensivity corrections) of Rydberg n-3s and n-3p States of the Smallest Ionized HCl-Water Cluster

Mariana G. Bezerra, Elizete Ventura, Silmar A. do Monte*

Departamento de Química, CCEN, Universidade Federal da Paraíba, 58059-900, João Pessoa, Brazil

*silmar@quimica.ufpb.br

**Contents**

1. Figure S1. Fragment-based analysis of the characters of the excited singlet states of the [H_3_O(H_2_O)_3_]^+^Cl^-^ ion-pair, computed at the MR-CISD level with B1 and B2.

2. Parameters used in the gradient program for the geometry optimization of the n-3s states at the MR-CISD level with B1 and B2.

3. Pairs of singlet and triplet states taken as single states (for B1 and B2), due to the degeneracies mentioned in the manuscript.

4. Electronic properties of the vertically excited states, obtained with B1 and B2.

4.1. Analysis of the Electronic properties and basis set effects for the vertically excited states, computed with B1 and B2.

5. Energetics of the two lowest lying dissociation channels, computed with B1 and B2.

6. Optimized geometries of the n-3s state, computed with B1 and B2.

7. Cartesian coordinates of the optimized geometries of the n-3s and ground ~~state~~ states, computed at the MR-CISD level with B1 and B2.

# 1. Characters of the excited states obtained with B1 and B2

(a)

(b)

Figure S1. Fragment-based analysis of the characters of the excited singlet states of the [H_3_O(H_2_O)_3_]^+^ Cl^-^ ion-pair, computed at the MR-CISD level with B1(a) and B2(b).

# 2. Parameters used in the gradient program for the geometry optimization of the n-3s states at the MR-CISD level with B1 and B2.

In the cigrdin file of COLUMBUS the standard values of the parameters rtol and dtol are 1e-6, while those of wndtol, wnatol and wnvtol are 1e-7. For the calculations performed in this work the first two parameters have been increased to 5e-6, while the latter three have been increased to 5e-7.

# 3. Pairs of singlet and triplet states taken as single states

**Table S1**. Pairs of singlet and triplet states taken as single states (for B1 and B2), due to the degeneracies mentioned in the text. The pairs of singlet and triplet states used for the calculation of ΔE_ST_ are also given.

|  | 6-31++G** (B1) | | | aug-cc-pVDZ(H ,Cl)/  dʹ-aug-cc-pVDZ(O) (B2) | | |
| --- | --- | --- | --- | --- | --- | --- |
| singlet states | combined states | | final state | combined states | | final state |
|  | S_0_ | -- | S_0_ʹ | S_0_ | -- | S_0_ʹ |
|  | S_1_ | -- | S_1_ʹ | S_1_ | -- | S_1_ʹ |
|  | S_2_ | S_3_ | S_2_ʹ | S_2_ | S_3_ | S_2_ʹ |
|  | S_4_ | S_5_ | S_3_ʹ | S_4_ | S_5_ | S_3_ʹ |
|  | S_6_ | S_7_ | S_4_ʹ | S_6_ | S_7_ | S_4_ʹ |
|  | S_8_ | S_9_ | S_5_ʹ | S_8_ | S_9_ | S_5_ʹ |
|  | S_10_ | S_11_ | S_6_ʹ | S_10_ | -- | S_6_ʹ |
|  | S_12_ |  | S_7_ʹ | S_11_ | S_12_ | S_7_ʹ |
| triplet states | combined states | | final state | combined states | | final state |
|  | T_1_ | T_2_ | T_1_ʹ | T_1_ | -- | T_1_ʹ |
|  | T_3_ | -- | T_2_ʹ | T_2_ | T_3_ | T_2_ʹ |
|  | T_5_ | T_6_ | T_3_ʹ | T_5_ | T_6_ | T_3_ʹ |
|  | T_4_ | T_9_ | T_4_ʹ | T_4_ | T_9_ | T_4_ʹ |
|  | T_7_ | T_8_ | T_5_ʹ | T_7_ | T_8_ | T_5_ʹ |
|  | T_10_ | -- | T_6_ʹ | T_10_ | -- | T_6_ʹ |
|  | T_11_ | T_12_ | T_7_ʹ | T_11_ | T_12_ | T_7_ʹ |
| pairs of states used for the calculation of ΔE_ST_ | S_1_ʹ/T_2_ʹ | | | S_1_ʹ/T_1_ʹ | | |
|  | S_2_ʹ/T_1_ʹ | | | S_2_ʹ/T_2_ʹ | | |
|  | S_3_ʹ/T_5_ʹ | | | S_3_ʹ/T_3_ʹ | | |
|  | S_4_ʹ/T_4_ʹ | | | S_4_ʹ/T_5_ʹ | | |
|  | S_5_ʹ/T_3_ʹ | | | S_5_ʹ/T_4_ʹ | | |
|  | S_6_ʹ/T_7_ʹ | | | S_6_ʹ/T_6_ʹ | | |
|  | S_7_ʹ/T_6_ʹ | | | S_7_ʹ/T_7_ʹ | | |

**4. Electronic properties of the vertically excited states, obtained with B1 and B2**.

**Table S2**. ΔE_v_, <r^2^>, μ, *y*, *f,* PR_NTO_*,* Ω, and ΔE_ST_ computed at the MR-CISD level for the first six excited singlet states. <r^2^> values for the ground and triplet states are also given, while μ and *y* of the ground state are also given. The results are shown for B1 and B2.

| basis set | state | ΔE_v_^a^ | main configurations^b^ | <r^2^>^c^ | μ^d^ | *y* | *f* (x10^2^) | PR_NTO_ | Ω | ΔE_ST_^a^ |
| --- | --- | --- | --- | --- | --- | --- | --- | --- | --- | --- |
| 6-31++G** (B1) | S_0_’ | 0.00 | 0.81cs^e^ | 86.49 | 4.57 | 0.0318 | --- | --- | --- | --- |
|  | S_1_’(1)^f^ | 6.12 (6.57)^g^ | 0.54n_z_3s + 0.30n_z_3p_z_ | 120.87 | 0.41 | 2.023 | 6.11 | 1.001 | 0.946 | 0.10 |
|  | S_2_’(2)^f^ | 6.13  (6.57)^g^ | 0.53n3s + 0.31n3p_z_ | 121.52 | 0.77 | 2.033 | 19.88 | 1.007 | 0.949 | 0.12 |
|  | S_3_’(3)^f^ | 6.91  (7.41)^g^ | 0.53n_z_3p | 135.71 | 4.60 | 2.140 | 5.84 | 1.444 | 0.949 | -0.02 |
|  | S_4_’(4)^f^ | 6.97  (7.48)^g^ | 0.41n3p + 0.29n_z_3p | 135.92 | 3.60 | 2.243 | 0.65 | 1.763 | 0.950 | 0.08 |
|  | S_5_’(5)^f^ | 7.06  (7.54)^g^ | 0.71n3p | 135.86 | 2.56 | 2.204 | 4.02 | 2.081 | 0.945 | 0.19 |
|  | S_6_’(6)^f^ | 7.54  (8.07)^g^ | 0.53n3p_z_ + 0.24n3s | 135.69 | 3.28 | 2.032 | 2.51 | 1.003 | 0.951 | 0.07 |
|  | S_7_’(7)^f^ | 7.56  (8.05)^g^ | 0.51n_z_3p_z_ + 0.29n_z_3s | 134.44 | 3.57 | 1.976 | 0.36 | 1.075 | 0.943 | 0.17 |
|  | T_1_’(2)^f^ | 6.01  (6.46)^g^ | 0.83n3s | 119.61 | --- | --- | --- | --- | --- | --- |
|  | T_2_’(1)^f^ | 6.02  (6.46)^g^ | 0.83n_z_3s | 119.24 | --- | --- | --- | --- | --- | --- |
|  | T_3_’(5)^f^ | 6.87 (7.38)^g^ | 0.83n3p | 135.38 | --- | --- | --- | --- | --- | --- |
|  | T_4_’(4)^f^ | 6.89  (7.41)^g^ | 0.43n_z_3p + 0.33n3p | 133.99 | --- | --- | --- | --- | --- | --- |
|  | T_5_’(3)^f^ | 6.93 (7.45)^g^ | 0.41n_z_3p + 0.27n3p | 135.92 | --- | --- | --- | --- | --- | --- |
|  | T_6_’(7)^f^ | 7.39  (7.92)^g^ | 0.83n_z_3p_z_ | 135.55 | --- | --- | --- | --- | --- | --- |
|  | T_7_’(6)^f^ | 7.47  (8.00)^g^ | 0.83n3p_z_ | 135.69 | --- | --- | --- | --- | --- | --- |
| aug-cc-pVDZ(H ,Cl)/  dʹ-aug-cc-pVDZ(O) (B2) | S_0_’ | 0.00 | 0.81cs^e^ | 84.55 | 4.72 | 0.034 | --- | --- | --- | --- |
|  | S_1_’(1)^f^ | 6.01 (6.64)^g^  (6.77)^h^ | 0.71n_z_3s + 0.12n_z_3p_z_ | 133.26 | 0.83 | 2.030 | 5.20 | 1.000 | 0.952 | 0.08 |
|  | S_2_’(2)^f^ | 6.03  (6.66)^g^  (6.79)^h^ | 0.70n3s | 133.78 | 0.94 | 2.034 | 17.6 | 1.004 | 0.954 | 0.10 |
|  | S_3_’(3)^f^ | 6.70  (7.41)^g^ | 0.64n_z_3p | 161.61 | 3.81 | 2.086 | 1.8 | 1.241 | 0.955 | 0.03 |
|  | S_4_’(4)^f^ | 6.74  (7.45)^g^ | 0.60n3p + 0.18n_z_3p | 161.95 | 1.35 | 2.289 | 0.8 | 1.919 | 0.956 | 0.03 |
|  | S_5_’(5)^f^ | 6.80  (7.50)^g^ | 0.79n3p | 162.14 | 1.09 | 2.205 | 1.1 | 2.123 | 0.953 | 0.11 |
|  | S_6_’(6)^f^ | 7.06  (7.77)^g^ | 0.67n_z_3p_z_ + 0.11n_z_3s | 172.47 | 6.09 | 1.991 | 0.6 | 1.122 | 0.949 | 0.09 |
|  | S_7_’(7)^f^ | 7.07  (7.80)^g^ | 0.70n3p_z_ | 174.27 | 6.26 | 2.033 | 1.0 | 1.005 | 0.956 | 0.04 |
|  | T_1_’(1)^f^ | 5.93  (6.55)^g^ | 0.82n_z_3s | 130.47 | --- | --- | --- | --- | --- | --- |
|  | T_2_’(2)^f^ | 5.93  (6.56)^g^ | 0.83n3s | 130.86 | --- | --- | --- | --- | --- | --- |
|  | T_3_’(3)^f^ | 6.67  (7.38)^g^ | 0.55n_z_3p + 0.26n3p | 158.67 | --- | --- | --- | --- | --- | --- |
|  | T_4_’(5)^f^ | 6.69 (7.40)^g^ | 0.82n3p | 159.01 | --- | --- | --- | --- | --- | --- |
|  | T_5_’(4)^f^ | 6.71 (7.42)^g^ | 0.42n3p + 0.28n_z_3p | 158.91 | --- | --- | --- | --- | --- | --- |
|  | T_6_’(6)^f^ | 6.97  (7.70)^g^ | 0.82n_z_3p_z_ | 170.60 | --- | --- | --- | --- | --- | --- |
|  | T_7_’(7)^f^ | 7.03  (7.77)^g^ | 0.83n3p_z_ | 171.31 | --- | --- | --- | --- | --- | --- |

^a^values in eV; ^b^n and 3p stand for the (n_x_,n_y_) and (3p_x_,3p_y_) pairs, respectively; ^c^values in au (a_0_^2^); ^d^values in Debye; ^e^cs = closed shell; ^f^The number in parenthesis identify the S/T pair with the most similar configurations, used to computed ΔE_ST_; ^g^values at the MR-CISD+P level; ^h^values at the MR-AQCC level; weights smaller than 0.1 have not been included.

**4.1. Analysis of the Electronic properties and basis set effects for the vertically excited states, computed with B1 and B2**.

The excitation energies obtained at the MR-CISD level for the singlet states are in the range from 6.12 to 7.56 and from 6.01 to 7.07 for B1 and B2, respectively (see Table S2). The size-extensivity correction (MR-CISD+P results in Table S2) increases the ΔE_v_ values considerably, by at least 0.44 eV. For the B1 and B2 basis sets, respectively, the ΔE_v_ values increase in the following ranges: 0.44 to 0.53 eV; 0.63 to 0.73 eV. As in the case of the TD-DFT/B3LYP results in ref. [19] the n-3s and n-3p states are well separated, at both MR-CISD and MR-CISD+P levels with B1 and B2. While at the TD-DFT level the separation is 0.60 eV, at the MR-CISD level the corresponding values are larger, especially for the smaller (B1) basis set. The n-3s and n-3p states are separated by 0.78 and 0.67 for B1 and B2, respectively (see Table S2). At the MR-CISD+P level these values increase to 0.84 and 0.75 eV. It is important to mention that, though significantly smaller than the basis set used for the TD-DFT/B3LYP calculations in ref. [19] (216 versus 287 Gaussian primitives), this latter basis set and the B2 basis set have similar accuracies. This conclusion comes from the fact that the TD-DFT/B3LYP results obtained with B2 are essentially the same as those shown in Figure 4(b) of ref. [19].

At the MR-CISD level the n-3p singlet states are in the ranges from 6.91 to 7.56 and 6.70 to 7.07 eV for B1 and B2, respectively. The largest differences concerning the ΔE_v_ values are observed for the highest n-3p singlet (S_7_’) and triplet (T_7_’) states, for B1 with respect to the other two basis sets. The ΔE_v_ value for S_7_’, computed with B1, is 0.49 and 0.56 eV higher than that obtained with B2 and B3, respectively (see Tables 2 and S2). At the MR-CISD+P level these differences decrease to 0.27 and 0.33 eV. The ranges obtained for the n-3p triplet states at the MR-CISD level are 6.87 to 7.47 and 6.67 to 7.03 for B1 and B2, respectively. The ΔE_v_ value for T_7_’, computed with B1, is 0.44 and 0.50 eV larger than that obtained with B2 and B3, respectively (see Tables 2 and S2). At the MR-CISD+P level these differences decrease to 0.23 and 0.29 eV. In summary, as can be seen from Tables 2 and S2 one has similar basis set effects for the singlet and triplet states, at both MR-CISD and MR-CISD+P levels.

At the MR-CISD+P/B2 level the ΔE_v_ values of the n-3s singlet states (S_1_’ and S_2_’) are 6.64 and 6.66 eV, very close to the highest-level MR-CISD+P/B3 results, 6.63 and 6.65 eV (see Tables 2 and S2). At the TD-DFT/B3LYP level they are much lower, at ~ 5.70 eV [19]. As discussed previously, the basis set used in this reference is similar in accuracy to B2. As the basis set effect is small for the n-3s states, the differences between the TD-DFT/B3LYP and both MR-CISD+P/B2 and MR-CISD+P/B3 results are very large. The results at the CASPT2 level with B1 are 6.46 and 6.52 eV [20], in very good agreement with our MR-CISD+P result (6.57 eV) obtained with the same basis set (see Table S2). Including the more accurate extensivity corrections at the MR-AQCC level, the ΔE_v_ values of S_1_’ and S_2_’ (computed with B2) increase 0.13 eV, as compared to the results at the MR-CISD+P level.

As mentioned in the main manuscript, in the case of the n-3p singlet states (S_3_’ to S_7_’) our highest-level results (again obtained at the MR-CISD+P level with the B3 basis set) are between 7.40 and 7.74 eV. With B2 and at the same computational level the range changes slightly, now between 7.38 and 7.77 eV (see Table 2). At the TD-DFT/B3LYP level these states are between 6.30 and 6.90 eV [19], again much lower than our MR-CISD+P results obtained with B2.

In the case of T_1_’ and T_2_’ the main configurations change as the basis set changes, that is, while for B1 these two states are characterized by the n3s and n_z_3s configurations, respectively, for B2 and B3 they undergo an interchange of configurations (see Tables 2 and S2). However, it is important to point out that these two states are virtually degenerate.

S_6_’ and S_7_’ are the n-3p singlet states whose main configurations change more with the basis set, as mentioned in the main manuscript (see also Tables 2 and S2). For B1 their main configurations are n3p_z_ and n_z_3p_z_, respectively (though with significant contributions of the n3s and n_z_3s configurations, see Table 2). However, for B2 and B3 their main configurations are swapped. Besides, the weights of the n3s and n_z_3s configurations become almost negligible for B2 and B3. Although the main configurations of S_4_’ (n3p and n_z_3p) do not change with the basis set, the relative weight of the n_z_3p configuration (the second most important one) decreases considerably, as one goes from B1 to the other basis sets (see Table 2 and S2).

As mentioned in the main manuscript, in the case of the n-3p triplet states the main configurations of T_3_’ to T_5_’ change with the basis set (see Tables 2 and S2). While for B1 n3p is the single dominant configuration for T_3_’, for B2 and B3 this configuration is the single dominant one for T_4_’ and T_5_’, respectively (see Table 2). For B1 T_4_’ and T_5_’ are characterized by a strong admixture between the n_z_3p and n3p configurations, while for B2 and B3 the same mixing is only obtained for T_3_’. The T_5_’ and T_4_’ states for B2 and B3 (respectively) are both characterized by a mixing between the n3p and n_z_3p configurations, but the relative weight of the second configuration is significantly higher for B2 (see Tables 2 and S2).

The largest value of ΔE_ST_ for B1 is 0.19 eV (obtained for the gap between S_5_’ and T_3_’, see Tables 2 and S2). For B2 and B3 it decreases to 0.11 and 0.09 eV, respectively. It is important to mention that there are discrepancies between the main configurations of some pairs of singlet and triplet states used to compute ΔE_ST_, especially for B1. For instance, for S_4_’ and T_4_’, the states which yield the value of 0.08 eV for this basis set, have n3p and n_z_3p as their main configurations, respectively (see Table S2). Besides, a negative value of -0.02 eV for ΔE_ST_ (between S_3_’ and T_5_’) has been obtained for B1. Only in this case a negative value for ΔE_ST_ has been obtained.

Looking more carefully at the main configurations in T_4_ʹ and T_5_ʹ it is clear that they are not only the same, but their weights are also very similar. The relative weights between their configurations, n_z_3p and n3p, are also very similar, that is, the ratio between these weights is ~ 1.30 and 1.52 for T_4_ʹ and T_5_ʹ, respectively (see Table S2). Thus, one important question arises, as a consequence of these similarities: should we use T_4_ʹ or T_5_ʹ to compute ΔE_ST_ for S_3_ʹ? The chosen criterion was the following: which one of these two triplet states has the *largest ratio between the weights* n_z_3p and n3p configurations (since S_3_ʹ is a n_z_3p state)? The answer to this question is T_5_ʹ, though for T_4_ʹ the mentioned ratio is very similar, as explained before. Consequently, the triplet states used to compute ΔE_ST_ for S_3_ʹ and S_4_ʹ are T_5_ʹ and T_4_ʹ, respectively, leading to the values of -0.02 eV and 0.08 eV. If the following criterion had been chosen instead: which one of these two triplet states has the *largest weight* for the n_z_3p configuration (since S_3_ʹ is a n_z_3p state)? In this case one would have to choose T_4_ʹ as the counterpart of S_3_ʹ and, consequently, T_5_ʹ as the counterpart of S_4_ʹ. These new pairs of states, S_3_ʹ/T_4_ʹ and S_4_ʹ/T_5_ʹ, lead to positive values for ΔE_ST_, 0.02 and 0.04 eV. In summary, the obtained negative value for ΔE_ST_ is a consequence of some degree of arbitrariness in the choice of the pairs used to compute ΔE_ST_. This arbitrariness is, in its turn, due to the nature of T_4_ʹ and T_5_ʹ in the case of the calculation with B1.

As mentioned in the main manuscript it is clear from Figures 2 and S1 that one has a large CR of the type Cl^-^→[H_3_O(H_2_O)_3_]^+^ for all studied excited singlet states. However, only for B3 this CR is consistent with the decrease of the dipole moments of all excited singlet states, as compared to that of the ground state. For B1 one has a slight increase for S_3_’, while for B2 the dipole moments of S_6_’ and S_7_’ increase more than 1D (see Tables 2 and S2).

The largest deviations of PR_NTO_ from the integer values of 1 and 2 are observed for S_3_’ and S_4_’, computed with B1 (see Tables 2 and S2).

**5. Energetics of the two lowest lying dissociation channels, computed with B1 and B2**

**Table S3**. Energies of the two dissociation channels (in eV) compared to that of the bonded ion pair. The zero-point energy (ZPE) corrections, computed at the MP2/aug-cc-pVTZ level, have been included.

| dissociation channel | MR-CISD | | MR-CISD + P | |
| --- | --- | --- | --- | --- |
|  | B1^a^ | B2^b^ | B1^a^ | B2^b^ |
| [H_3_O(H_2_O)_3_]^+^ + Cl^-^ | 4.28 | 4.43 | 4.56 | 4.70 |
| [H_3_O(H_2_O)_3_] + Cl | 4.49 | 4.65 | 4.91 | 5.19 |

^a^B1: 6-31++G**, ^b^B2: aug-cc-pVDZ(H,Cl)/dʹ-aug-cc-pVDZ(O)

In Table S3 the energies of the ionic and neutral channels (computed with B1 and B2) are compared to that of the bonded ion pair. The studied ion pair is strongly bonded by 4.28 to 4.70 eV, depending on the computational level and on the basis set. The extensivity correction (+P) increases the binding energies by 0.28 and 0.27 eV for B1 and B2, respectively.

**6. Optimized geometries of the n-3s state, computed with B1 and B2**.

Surprisingly, the same optimized geometry is a stationary point for S_1_, S_2_ and S_3_ (for a given basis set). The geometries obtained at the MR-CISD level with B1 and B2 are shown in Figure S2. Their cartesian coordinates are given in Table S4. As can be seen from Figure S2, one has a type of asymmetrically pre-dissociated structure with respect to the release of Cl. The “intermolecular” distances (that is, those between H_3_O(H_2_O)_3_ and Cl fragments) show much larger variations than the distances within the H_3_O(H_2_O)_3_ fragment, as compared to those of the ground state structure. For instance, the H bond distances between H_3_O and the water molecules increase 0.053 and 0.057 Å, for B1 and B2, respectively, while the OCl distance increases 16.845 and 16.803 Å for these two basis sets. For B1 the H•••Cl distance increases in a range varying from 15.770 to 16.783 Å, while for B2 the corresponding range is 15.770 to 16.682 Å. The behavior of the OH bond distance of the H_3_O moiety shows a qualitative difference between B1 and B2, that is, while for the former basis set it decreases 0.026 Å for the latter it essentially does not change. However, such analysis should be taken with caution, as the ground state and the n-3s geometry have been optimized with different methods and basis sets.

If the ground state is optimized at the same levels as those of n-3s the H bond distances between H_3_O and the water molecules decrease 0.015 and 0.019 Å, for B1 and B2, respectively, while the OCl distance increases 16.879 and 16.807 Å for these two basis sets. The H•••Cl distance increases in a range varying from 15.646 to 16.661 Å for B1, while for B2 the corresponding range is 15.681 to 16.682 Å. The OH bond distance of the H_3_O moiety almost does not change for both basis sets. The cartesian coordinates of the ground state geometry are given in Table S5.

At the optimized geometries S_0_ to S_2_ are n-3s states, while S_3_ is the ionic state. The first three states are essentially degenerate, while S_3_ is 0.21 and 0.15 eV higher at the MR-CISD/B1 and MR-CISD/B2 levels, respectively. Upon inclusion of +P extensivity correction these values change to 0.06 and 0.28 eV.

**Figure S2.** Optimized structure for the singlet n-3s states (S_1_, S_2_ and S_3_) obtained at the MR-CISD level with the B1 and B2 basis sets. The main geometrical parameters are given in plot. The values in parenthesis have been obtained with B2.

# 7. Cartesian coordinates (in Bohr) for the optimized geometries of the n-3s and ground states

As explained in the manuscript the obtained geometries of the three n-3s singlet states are the same, for a given basis set.

**Table S4.** Cartesian coordinates (in Bohr) for the optimized geometries of the n-3s states

| B1 | B2 |
| --- | --- |
| Cl -33.77607572 -9.50545919 -4.89509733  O 3.19515830 0.02775731 0.01159952  O 0.87496306 4.10426024 -1.25018216  O 0.92662098 -3.11414848 -2.91683908  O 0.87983283 -0.94826831 4.16842577  H 2.48528357 1.71866237 -0.45152056  H 2.50692241 -1.22218527 -1.22989861  H 2.48803134 -0.42592830 1.70620940  H 0.91184122 5.63209378 -0.28464543  H -0.87291551 3.60011483 -1.35028790  H 0.98659677 -3.04341619 -4.72218204  H -0.82821142 -2.95711794 -2.45306129  H 0.93620408 -2.54776001 5.00887244  H -0.87161011 -0.63247850 3.77859208 | Cl -34.12073515 -8.06087706 -3.73910665  O 3.25463735 -0.01220359 -0.00515852  O 0.97374844 4.10087255 -1.26427296  O 0.85781612 -3.10379175 -2.90361594  O 0.89657721 -0.92172331 4.15610497  H 2.54743922 1.68375955 -0.45395377  H 2.50041273 -1.23149333 -1.23909368  H 2.51430960 -0.45374727 1.67856799  H 0.94598971 5.54796503 -0.18136891  H -0.76301879 3.56354465 -1.38631482  H 0.80784452 -2.88612928 -4.69741720  H -0.86648938 -2.90069667 -2.35081887  H 0.81945248 -2.58188332 4.86706737  H -0.82524357 -0.50563800 3.72924926 |

**Table S5.** Cartesian coordinates (in Bohr) for the optimized geometries of the ground state

| B1 | B2 |
| --- | --- |
| Cl -3.28372429 0.07610273 -0.01263177  O 3.31230443 -0.00869999 0.02089888  O 0.87283417 3.96607013 -1.18519571  O 0.79138785 -2.97927104 -2.80543650  O 0.79138785 -2.97927104 -2.80543650  H 2.61559808 1.70000873 -0.40823629  H 2.57851537 -1.21777664 -1.24113304  H 2.57631612 -0.47901689 1.70221799  H 0.83602723 5.59898287 -0.43266182  H -0.78438006 3.20361721 -0.96294699  H 0.74484878 -3.13869135 -4.59612900  H -0.85001123 -2.36713201 -2.24941780  H 0.70615982 -2.37630983 5.05835951  H -0.85596278 -0.69746336 3.21820861 | Cl -3.29830671 0.04746993 -0.04833094  O 3.35509701 -0.00476398 0.02438607  O 0.83859013 3.91173087 -1.22874362  O 0.80207430 -2.99041101 -2.76412452  O 0.74385686 -0.87131578 3.98354139  H 2.61335857 1.68555451 -0.41017926  H 2.59668949 -1.21047313 -1.22892297  H 2.57344642 -0.47030831 1.68826369  H 0.73015986 5.48506384 -0.36524119  H -0.78643509 3.07707908 -0.99718579  H 0.71237619 -3.02317990 -4.55960434  H -0.81543958 -2.33762339 -2.17309343  H 0.61197737 -2.40577052 4.91136034  H -0.86074366 -0.65274840 3.10678670 |
